# Machine Learning Methods for the Diagnosis of Chronic Obstructive Pulmonary Disease in Healthy Subjects: Retrospective Observational Cohort Study

**DOI:** 10.2196/24796

**Published:** 2021-07-06

**Authors:** Shigeo Muro, Masato Ishida, Yoshiharu Horie, Wataru Takeuchi, Shunki Nakagawa, Hideyuki Ban, Tohru Nakagawa, Tetsuhisa Kitamura

**Affiliations:** 1 Department of Respiratory Medicine Nara Medical University Nara Japan; 2 Department of Respiratory and Immunology, Medical AstraZeneca KK Osaka Japan; 3 Department of Data Science, Medical AstraZeneca KK Osaka Japan; 4 Center for Technology Innovation–Artificial Intelligence, Research & Development Group Hitachi, Ltd Tokyo Japan; 5 Hitachi Health Care Center Hitachi, Ltd Ibaraki Japan; 6 Division of Environmental Medicine and Population Sciences Department of Social and Environmental Medicine Graduate School of Medicine, Osaka University Osaka Japan

**Keywords:** chronic obstructive pulmonary disease, airflow limitation, medical check-up, Gradient Boosting Decision Tree, logistic regression

## Abstract

**Background:**

Airflow limitation is a critical physiological feature in chronic obstructive pulmonary disease (COPD), for which long-term exposure to noxious substances, including tobacco smoke, is an established risk. However, not all long-term smokers develop COPD, meaning that other risk factors exist.

**Objective:**

This study aimed to predict the risk factors for COPD diagnosis using machine learning in an annual medical check-up database.

**Methods:**

In this retrospective observational cohort study (ARTDECO [Analysis of Risk Factors to Detect COPD]), annual medical check-up records for all Hitachi Ltd employees in Japan collected from April 1998 to March 2019 were analyzed. Employees who provided informed consent via an opt-out model were screened and those aged 30 to 75 years without a prior diagnosis of COPD/asthma or a history of cancer were included. The database included clinical measurements (eg, pulmonary function tests) and questionnaire responses. To predict the risk factors for COPD diagnosis within a 3-year period, the Gradient Boosting Decision Tree machine learning (XGBoost) method was applied as a primary approach, with logistic regression as a secondary method. A diagnosis of COPD was made when the ratio of the prebronchodilator forced expiratory volume in 1 second (FEV_1_) to prebronchodilator forced vital capacity (FVC) was <0.7 during two consecutive examinations.

**Results:**

Of the 26,101 individuals screened, 1213 met the exclusion criteria, and thus, 24,815 individuals were included in the analysis. The top 10 predictors for COPD diagnosis were FEV_1_/FVC, smoking status, allergic symptoms, cough, pack years, hemoglobin A_1c_, serum albumin, mean corpuscular volume, percent predicted vital capacity, and percent predicted value of FEV_1_. The areas under the receiver operating characteristic curves of the XGBoost model and the logistic regression model were 0.956 and 0.943, respectively.

**Conclusions:**

Using a machine learning model in this longitudinal database, we identified a number of parameters as risk factors other than smoking exposure or lung function to support general practitioners and occupational health physicians to predict the development of COPD. Further research to confirm our results is warranted, as our analysis involved a database used only in Japan.

## Introduction

Chronic obstructive pulmonary disease (COPD) is characterized by airflow limitation associated with persistent respiratory symptoms. Most patients with COPD experience exacerbation of symptoms and are at high risk of developing comorbidities such as cardiovascular disease [1].

Long-term exposure to tobacco smoke, vapor, gas, dust, and fumes is an established major risk factor for COPD [2]. However, only a small percentage of smokers develop airflow limitation, while nonsmokers can develop COPD [3]. These inconsistencies indicate that risk factors other than long-term smoking are associated with COPD [4].

The prevalence of COPD has been reported to be 12% to 13% among smokers [5]. However, only 9.4% of patients with airflow limitation have a previous diagnosis of COPD, and European data indicate that up to 80% of COPD cases are undiagnosed [6], suggesting delays in the diagnosis of COPD. The ARCTIC observational cohort study showed that late COPD diagnosis was associated with a higher exacerbation rate and increased comorbidities and costs compared with early diagnosis [7].

To address the issue of undiagnosed COPD, significant risk factors for airflow limitation other than smoking should be identified and evaluated in routine clinical practice. In a cohort study of 9040 individuals from the Japanese general population, concomitant *Chlamydia pneumoniae* and *Mycoplasma pneumoniae* seropositivity was found to be an independent risk factor for airflow limitation [8]. Additionally, Sato et al employed an annual health examination with pulmonary function tests measuring airflow limitation to identify undiagnosed patients with COPD among the Japanese population and found that iron deficiency might be associated with COPD development [9]. However, the follow-up duration of these cohorts was short (<3 years), limiting their ability to identify risk factors for COPD in the general population.

A large questionnaire-based surveillance demonstrated some improvement in diagnostic rates for COPD; however, approximately 60% of eligible participants failed to respond to the questionnaire [10]. While these results suggest that identifying robust and relevant risk factors is likely to improve early diagnosis, the slow progression and heterogeneity of the disease have hindered the identification of such risk factors for COPD development.

The recently reported “Subtype and Stage Inference” machine learning computational model identified subtypes of patients with COPD [11]. Compared with traditional approaches, the advantages of machine learning include the ability to process complex nonlinear relationships between predictors and to provide novel outputs. Therefore, the aim of this study was to apply machine learning methods to predict possible risk factors for the development of airflow limitation, an essential feature of COPD diagnosis, using a Japanese medical check-up database comprising data from a number of healthy subjects to support the early diagnosis of COPD by general practitioners and occupational health physicians.

## Methods

### Study Design and Population

This was a retrospective observational cohort study to predict the risk factors for COPD diagnosis in healthy individuals. The analysis data set comprised individuals aged ≥30 years who had undertaken more than two medical check-ups, had no history of lung cancer or asthma at the first medical check-up, and could be classified as either having a diagnosis of COPD or as not having COPD. This study was designed according to the *Transparent Reporting of a Multivariate Prediction Model for Individual Prognosis or Diagnosis* guidelines for prognostic studies [12].

The study protocol was reviewed and approved by the ethics committee of MINS (a nonprofit organization in Tokyo, Japan) and the Research & Development Group and Corporate Hospital Group of Hitachi, Ltd (Tokyo, Japan) prior to the start of data analysis. Individual informed consent was obtained using an opt-out model in agreement with the Institutional Review Board at Hitachi, Ltd. This study was conducted in accordance with the ethical principles of the Declaration of Helsinki.

### Data Source

The data source was annual medical check-up data for all Hitachi employees from April 1998 to March 2019. Data were archived in a high-security server that was managed with limited access rights by Hitachi. The annual medical check-up includes clinical measurements and questionnaires to examine the health of employees (Multimedia Appendix 1). Such questionnaires are utilized by Japanese organizations to evaluate their employees’ health and give advice about health promotion, such as giving up smoking and exercising regularly based on the second term of the National Health Promotion Movement in the 21st century (Health Japan 21) issued by the Ministry of Health, Labour, and Welfare in Japan [13].

### Definition of COPD

COPD was considered according to the lung function status at two consecutive measurements during an annual lung function test when the prebronchodilator (pre-BD) forced expiratory volume in 1 second/forced vital capacity (FEV_1_/FVC) was <0.7, as previously employed in a large population-based cohort study [14]. Individuals having a pre-BD FEV_1_/FVC ≥0.7 in at least three consecutive annual lung function test measurements were classified as non-COPD. For individuals with more than three records in the non-COPD group, the most recent three records were analyzed. Individuals having less than two lung function tests were excluded from all analyses. Spirometry was calibrated and performed by trained paramedical personnel according to the American Thoracic Society/European Respiratory Society guidelines [15,16].

### Statistical Analysis

#### Age at COPD Diagnosis

The age distribution for disease diagnosis was evaluated and stratified by smoking status (current smoker, exsmoker, or nonsmoker). The age at COPD diagnosis was defined as the age at the first of two consecutive measurements in which the pre-BD FEV_1_/FVC was <0.7.

#### Risk Factor Prediction Using Machine Learning

Two types of models were constructed for predicting the risk factors for COPD diagnosis within 3 years as follows: a machine learning method (Gradient Boosting Decision Tree machine learning [XGBoost] [17]) and an established statistical method (logistic regression [18]). Individuals who did not meet the study inclusion criteria and/or had lung cancer/asthma were excluded from the analyses. Any individuals with missing data during the 3 years prior to the diagnosis year in the COPD group or during the most recent 3 years in the non-COPD group were excluded from the analyses. Propensity scores were calculated based on age, sex, smoking status, BMI, eosinophil count (EOS), and FEV_1_.

Data were randomly divided into a training data set and a test data set at a ratio of 7:3, with the same ratio of COPD to non-COPD individuals. Propensity scoring was used to balance the characteristics of COPD and non-COPD individuals (caliper: 0.2) in the training and test data sets. Next, the training data set was randomly divided 8:2 for model construction (XGBoost and logistic) and evaluation of model performance, respectively. The data split, model construction, and evaluation processes were repeated five times for cross-validation (5-CV approach) [19]. Model parameters, including the depth of the tree and regularization factor, were refined during performance evaluation by the 5-CV approach. Finally, the most optimal model was generated by applying the best parameters confirmed by the 5-CV approach. To evaluate model performance in the unlearned data, the most optimized model was used to evaluate the test data set.

Model construction by logistic regression was performed in a similar way to the XGBoost method. Models were constructed in the training data set (randomly sampled data from the entire data set) and subsequently validated in the test data set after model evaluation.

Following model construction, the area under the receiver operating characteristic curve (AUC), positive predictive value, sensitivity, specificity, and F1-measure were calculated for each model to evaluate the performance under both 5-CV and test conditions [20]. The feature importance of the machine learning model was calculated to examine the contribution of each predictor to the model constructed using the Gini impurity method [19]. The feature weight of the logistic regression model was also calculated. All analyses were performed using Python 3.6 software (Python Software Foundation).

## Results

### Individuals

**Figure 1:**
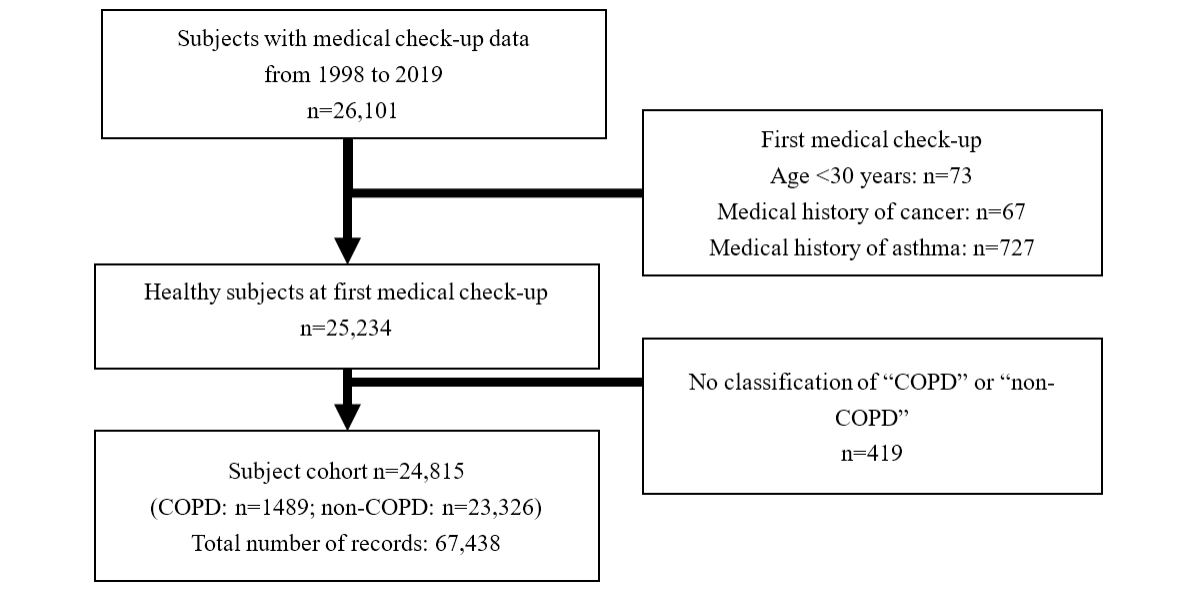
Flow diagram of the study. COPD: chronic obstructive pulmonary disease.

Data from 26,101 individuals (employees and their families) aged 30 to 75 years, who underwent annual check-ups between April 1998 and March 2019 were included in our analysis. The total number of medical check-up records was 318,568. All 26,101 individuals had lung function test measurements for 3 consecutive years. The medical records for 73 individuals aged <30 years at the first medical check-up, 67 individuals with a history of cancer, and 727 individuals with a history of asthma were excluded, as were data from 419 individuals who had already been diagnosed with COPD (subjects for whom all data points of pre-BD FEV_1_/FVC were <0.7 during the observational period) or had not been classified as either COPD or non-COPD (subjects with pre-BD FEV_1_/FVC <0.7 without two consecutive measurements). Accordingly, data for 24,815 individuals (corresponding to 67,438 records) were included in the analyses (Figure 1).

### Baseline Characteristics

Table 1 shows the baseline characteristics of the COPD and non-COPD groups. Overall, 1489 individuals were considered as having COPD (pre-BD FEV_1_/FVC <0.7 at two consecutive measurements during annual lung function tests). In comparison with the non-COPD group, the COPD group had a lower BMI, worse lung function (pre-BD FEV_1_, pre-BD percent predicted value of FEV_1_ [%FEV_1_], and pre-BD FEV_1_/FVC), and greater emphysematous change and chronic inflammation as determined by computed tomography. Furthermore, comorbidities, such as arrythmia, duodenal ulcer, colorectal polyp, angina, stomach ulcer, and kidney disease, were more prevalent in the COPD group. Statistically significant differences in hematological parameters (mean corpuscular volume [MCV], mean corpuscular hemoglobin concentration [MCHC], mean corpuscular hemoglobin [MCH], hemoglobin [Hb], and hematocrit [HT] [15]) between the COPD and non-COPD groups were also observed. Inflammatory markers, particularly white blood cell (WBC) count and EOS, were also significantly higher in the COPD group.

**Table 1:** Subject characteristics stratified by chronic obstructive pulmonary disease status.

Characteristic		Non-COPD^a^ (n=23,326)	COPD (n=1489)	*P* value
Age (years), mean (SD)		42 (9.1)	48 (9.3)	<.001
Female, n (%)		3841 (16.5%)	58 (3.9%)	<.001
**Smoking status, n (%)**				<.001
	Current smoker	10,632 (45.6%)	1,021 (68.6%)	
	Exsmoker	3534 (15.2%)	202 (13.6%)	
	Nonsmoker	9153 (39.3%)	266 (17.9%)	
	Unknown/missing	7 (0.0%)	0 (0.0%)	
BMI (kg/m^2^), mean (SD)		23 (3.2)	22 (2.7)	<.001
**Lung function test, mean (SD)**				
	Prebronchodilator FEV_1_^b^	3.4 (0.7)	3.1 (0.6)	<.001
	Prebronchodilator FVC^c^	4.1 (0.8)	4.2 (0.8)	<.001
	Prebronchodilator FEV_1_/FVC	83.7 (5.4)	74.9 (5.1)	<.001
**Comorbidity, n (%)**				
	Arrythmia	107 (0.5%)	16 (1.1%)	.003
	Duodenal ulcer	158 (0.7%)	19 (1.3%)	.02
	Colorectal polyp	43 (0.2%)	13 (0.9%)	<.001
	Angina	56 (0.2%)	10 (0.7%)	.006
	Stomach ulcer	180 (0.8%)	29 (1.9%)	<.001
	Kidney disease	77 (0.3%)	12 (0.8%)	.01
**Computed tomography finding, n (%)**				
	Bulla, bleb	108 (0.5%)	31 (2.1%)	<.001
	Moderate emphysema	18 (0.1%)	13 (0.9%)	<.001
	Mild emphysema	96 (0.4%)	27 (1.8%)	<.001
	Calcification of left anterior descending coronary artery	128 (0.5%)	16 (1.1%)	.02
	Chronic inflammation	342 (1.5%)	43 (2.9%)	<.001
**Laboratory parameters, mean (SD)**				
	Albumin (U/L)	4.4 (0.2)	4.3 (0.2)	<.001
	Alanine aminotransferase (U/L)	209.5 (53.7)	215.2 (54.6)	<.001
	Aspartate aminotransferase (U/L)	26.4 (14.8)	24.3 (12.8)	<.001
	Blood urea nitrogen (mg/dL)	14.1 (3.2)	14.7 (3.3)	<.001
	Cholinesterase (U/L)	320.8 (60.1)	307.8 (58.8)	<.001
	Estimated glomerular filtration rate (mL/min/1.73 m^2^)	83.6 (14.6)	80.2 (14.1)	<.001
	Eosinophil count (cells/mm^3^)	183.2 (124.5)	195.4 (125.9)	<.001
	Gamma-glutamyl transferase (U/L)	42.7 (34.4)	45.8 (34.5)	<.001
	Hemoglobin (g/dL)	14.7 (1.4)	14.9 (1.1)	<.001
	Hemoglobin A_1c_ (%)	5.3 (0.7)	5.4 (0.7)	<.001
	Hematocrit (%)	44.0 (3.6)	44.6 (3.1)	<.001
	MCH^d^ (pg)	30.5 (1.8)	31.1 (1.7)	<.001
	MCHC^e^ (g/L)	33.4 (1.0)	33.3 (0.8)	<.001
	MCV^f^ (fL)	91.3 (4.6)	93.4 (4.4)	<.001
	WBC^g^ count (×10^2^ cells/µL)	58.8 (15.0)	62.8 (15.5)	<.001

^a^COPD: chronic obstructive pulmonary disease.

^b^FEV_1_: forced expiratory volume in 1 second.

^c^FVC: forced vital capacity.

^d^MCH: mean corpuscular hemoglobin.

^e^MCHC: mean corpuscular hemoglobin concentration.

^f^MCV: mean corpuscular volume.

^g^WBC: white blood cell.

### Percentage of Individuals With COPD

**Figure 2:**
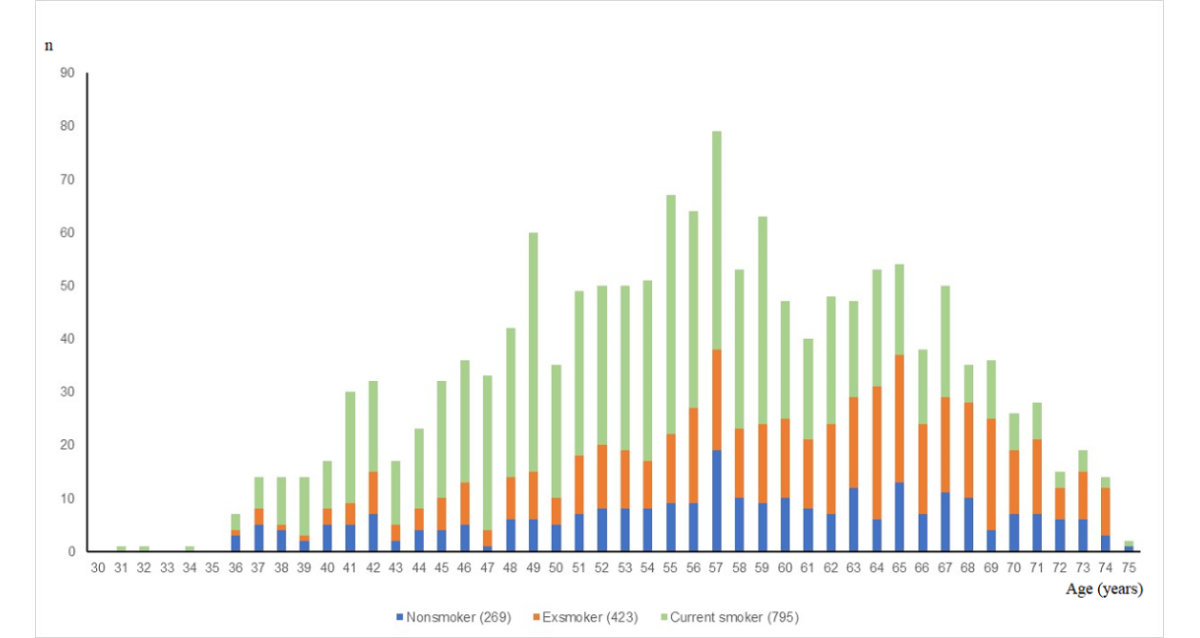
Diagnostic age for chronic obstructive pulmonary disease (COPD) according to smoking status.

The overall percentage of individuals with COPD was 6.0% (1489/24,815). According to smoking status, the percentage of individuals with COPD was 8.8% (1021/11,653) among current smokers and 5.4% (202/3736) among exsmokers. Notably, 2.8% (266/9419) of nonsmokers had developed COPD. The peak age at diagnosis of COPD among current smokers and exsmokers was 55 years and 65 years, respectively (Figure 2).

### Risk Factors for COPD Diagnosis

Overall, 20,265 individuals (COPD: n=954; non-COPD: n=19,311) with 51,432 records (COPD: n=2435; non-COPD: n=48,997) out of 24,815 individuals who met the criteria (Multimedia Appendix 2) were included in the machine learning analysis. Table 2 shows the model performance of the XGBoost and logistic regression models. For both models, the AUC, accuracy, sensitivity, specificity, and F-measure were generally similar between the training and test data sets. The XGBoost model had a higher positive predictive value (0.505) than the logistic regression model (0.441). The AUC was high in the training and test sets for both models (range: 0.892-0.956). Additionally, the accuracy and specificity exceeded 0.883 and 0.879, respectively, for both models.

The most important predictive factors for COPD diagnosis were lung function tests (ie, FEV_1_/FVC, percent vital capacity [%VC], and %FEV_1_) and smoking status, followed by cough, hematological indices (ie, MCV, MCHC, MCH, Hb, and HT), treatment with antidiabetic drugs, hemoglobin A_1c_, serum albumin, total protein, and BMI. Other predictive risk factors were EOS, serum alanine aminotransferase, WBC count, and urinary WBC count (Table 3). Logistic regression analysis showed that low FEV_1_/FVC and %FEV_1_; high %VC; high MCV, MCHC, and Hb; and low HT and MCH were related factors, and that individuals treated with antidiabetic drugs had a higher number of associated risk factors for COPD. Low serum albumin, low total protein, and low BMI were also confirmed as risk factors (Multimedia Appendix 3).

**Table 2:** Comparison of performance of the Gradient Boosting Decision Tree machine learning (XGBoost) and logistic regression models.

Variable	XGBoost^a^ model			Logistic regression model	
	Training, mean (SE)	Test, mean	Training, mean (SE)		Test, mean
Positive predictive value	0.505 (0.099)	0.362	0.441 (0.110)		0.285
AUC^b^	0.956 (0.015)	0.898	0.943 (0.022)		0.892
Accuracy	0.917 (0.032)	0.918	0.884 (0.049)		0.883
Sensitivity	0.845 (0.021)	0.877	0.874 (0.039)		0.901
Specificity	0.960 (0.016)	0.919	0.946 (0.025)		0.882
F-measure	0.370 (0.107)	0.513	0.306 (0.110)		0.434

^a^XGBoost: Gradient Boosting Decision Tree machine learning.

^b^AUC: area under the receiver operating characteristic curve.

**Table 3:** Importance of each predictor in the XGBoost model.

Variable	Importance value
Forced expiratory volume in 1 second/forced vital capacity	0.2824
Smoking status	0.0329
Allergic symptoms (yes/no)	0.0303
Symptom-cough (yes/no)	0.0294
Smoking-pack year	0.0222
Hemoglobin A_1c_	0.0197
Albumin	0.0195
Mean corpuscular volume	0.0177
%Vital capacity	0.0165
%Forced expiratory volume in 1 second	0.0164
Treatment with an antidiabetic drug (yes/no)	0.0162
Allergic disease (yes/no)	0.0146
Hematocrit	0.0144
Urinary red blood cells	0.0143
Hemoglobin	0.0138
Age	0.0128
Smoking duration	0.0127
High density lipoprotein cholesterol	0.0123
Mean corpuscular hemoglobin concentration	0.0122
Total protein	0.0118
BMI	0.0118
Number of eosinophils	0.0115
Mean corpuscular hemoglobin	0.0114
Serum white blood cells	0.0111
Fasting blood sugar	0.0110
Serum alanine aminotransferase	0.0108
Pulse rate	0.0108
Forced expiratory volume in 1 second	0.0107
Urinary white blood cells	0.0104
Diastolic blood pressure	0.0103

For future utilization of risk factors for disease assessment in daily clinical practice, the machine learning process was validated using a questionnaire to predict risk factors for COPD development (Multimedia Appendix 1). Of 30 variables, 25 were clinical parameters that overlapped between the two methods. The top 30 risk factors also included the following five questions: “I am regularly doing exercise,” “I have chest compression and pain,” “Average sleeping time in the past 1 month,” “I have breakfast every day,” and “Body fat ratio” (Multimedia Appendix 4). Among these, logistic regression analysis showed that insufficient sleeping time and not having breakfast every day were risk factors for COPD (Multimedia Appendix 3).

## Discussion

This study applied a machine learning method, a powerful tool to analyze large quantities of complex data, to predict risk factors for COPD. This is the first study to investigate more than 300,000 records from working-age adults in Japan utilizing an annual medical check-up database. This system allows healthy employees to track their health conditions over time by clinical measurements and questionnaires. We found that the most significant predictor of COPD diagnosis was the absolute value of FEV_1_/FVC, indicating that low FEV_1_ in early adulthood is an important factor in the development of COPD. Childhood asthma is associated with impaired lung function, lower lung function in adulthood, and higher risk of COPD even for nonsmoker participants, as previously reported by Martinez et al [21]. In our speculation, some part of the nonsmoker COPD population might have had a history of childhood asthma, increasing susceptibility to passive smoke exposure or airway pollution and resulting in the early diagnosis of COPD in nonsmokers compared with exsmokers in the study. Smoking status had the second highest impact on disease diagnosis. Among individuals with a smoking history, the peak age of COPD diagnosis was older in exsmokers than in current smokers. This finding suggests that smoking cessation delays the diagnosis of COPD, consistent with a previous study in which smoking cessation was reported to affect the natural history of COPD [22].

Erythrocyte indices (MCV and MCHC) might also be available as potential predictors of COPD diagnosis in addition to lung function measurements. These data are supported by a previous report in which continuous smoking had a significant effect on hematological parameters compared with nonsmoking, and it may be associated with an increased risk of COPD [23]. The increased levels of MCV and MCHC in individuals with COPD support a previous finding that impaired lung function has a strong association with ischemic heart disease [24]. Conversely, the presence of an allergic disease appeared to have a preventive effect on airflow limitation, which is in contrast with observations from the Tasmanian Longitudinal Health Study in which the presence of allergic diseases was an early predictor of lung trajectories toward COPD [25]. However, the Hokkaido cohort study showed that subjects with multiple asthma-like features had slower lung function decline [26]. From the findings of our observational study in Japan, we can speculate that early diagnosis and intervention for allergic diseases may have less impact on lung function and that regular and frequent medical intervention could lead to an overall increase in life expectancy among patients who can readily access appropriate treatment by respiratory specialists.

Furthermore, individuals with decreased levels of serum albumin and total protein, as well as lower hemoglobin A_1c_ and BMI may be at risk of developing cachexia, a common condition among patients with COPD [27]. With respect to other identified risk factors, a retrospective cross-sectional study showed an association between EOS and airflow limitation in patients with COPD [28]. Given that increased alanine aminotransferase levels have been observed in patients with obstructive sleep apnea [29], individuals at risk of developing COPD might be exposed to intermittent hypoxia, indicating that a reduced sleeping time, as determined in the study questionnaire, might also represent a risk factor for COPD. Even minor changes in hematological parameters might be attributable to hypoxic conditions, leading to sleep disruption. Additionally, frequently missing breakfast might accelerate malnutrition in the COPD group. Furthermore, significantly higher prevalence rates of chronic neck and lower back pain in patients with COPD compared with healthy individuals were observed in a population-based study, although the findings were not confirmed by logistic regression analysis [30], and the link between COPD and back pain remains unknown. The observation of increased WBC counts in patients with COPD compared with healthy controls [31] suggests that systemic inflammation may be involved in the pathogenesis of COPD [32].

Our results also indicate that smoking cessation should be prioritized for the prevention of COPD and that smokers with sleep disturbances, back pain, and/or low BMI and malnutrition may be at increased risk of developing COPD and should be considered as candidates for lifestyle intervention therapy. Furthermore, the five key questions included in our questionnaire should be validated in future investigations and potentially implemented in daily practice as part of an annual medical check-up to prevent COPD.

The positive predictive value of the XGBoost model was comparable to that of a self-scored persistent airflow obstruction screening questionnaire in the Japanese population previously reported by Samukawa et al [33]. However, our models showed more accuracy because the sensitivity and specificity of our models achieved higher figures, and the AUC reached over 0.9 compared with that of the questionnaire, which ranged from 0.595 to 0.612. The AUCs of the XGBoost and logistic regression models were similar, while the most important factor related to COPD diagnosis was FEV_1_ in both models. However, some variables differed in importance in each model. Kuhn et al reported that machine learning approaches can incorporate high-order nonlinear interactions among predictors that cannot be addressed by traditional modeling approaches (eg, logistic regression models) [34]. However, machine learning methods cannot elucidate whether a causal relationship exists between the identified variable and the disease. Thus, the association between risk factors detected using a machine learning model and COPD requires validation in future prospective studies.

A strength of this study was the use of longitudinal lung function test data from healthy individuals from April 1998 to March 2019. In general, medical checkup data are not linked to medical records, meaning that profiles of lung function tests over time could not be investigated. However, it was possible to evaluate longitudinal lung function tests because the database included data from individuals from a point in time when they were healthy until they had developed COPD. Additionally, data from healthy individuals were included, allowing lung function test results from when they were diagnosed with COPD to be investigated. Finally, both clinical measurements and questionnaire variables were included in the database, thereby increasing the potential to identify several different risk factors for COPD.

The limitations of this study include the definition of COPD diagnosis by airflow limitation with pulmonary function tests. Instead of post-BD spirometry data as suggested by the ATS/European respiratory guidelines, we employed pre-BD spirometry data for the diagnosis of COPD since no post-BD spirometry was performed in the annual medical check-up. The precise diagnosis of COPD cannot always be demonstrated by airflow limitation alone; however, we believe that the diagnostic approach was reasonable from a clinical perspective as airflow limitation has been reported to be a poor prognostic factor in the general population [35]. Low lung function values (FEV_1_/FVC <0.7) might be observed at a single time point in some individuals for no discernable reason. Therefore, we considered COPD as lung function of FEV_1_/FVC <0.7 on two consecutive occasions. In terms of differentiation between asthma and COPD, we cannot exclude the possibility of misclassification of asthma as COPD in some patients since reversibility tests were not performed in the annual medical check-up, but participants with a medical history of asthma were excluded. Additionally, a database from a single organization was analyzed in this study; thus, the results might include bias based on the type of industry or the organizational structure of the company, limiting the generalizability of the findings. To obtain more generalizable findings, studies using other databases are necessary. Finally, some unknown confounders may have remained; therefore, we plan to perform model validation by analyzing other databases. Well-controlled prospective studies should be conducted to confirm the predictive factors for COPD diagnosis.

In conclusion, our machine learning method applied to longitudinal medical check-up data, including general questionnaires and laboratory parameters, identified hematological, nutritional, and inflammatory parameters as potential risk factors for COPD. These parameters, along with lung function and smoking status, may be useful in identifying at-risk individuals and may lead to an earlier diagnosis.

## Appendix

Multimedia Appendix 1 Clinical assessments and questions used in the analysis, and list of variables.

Multimedia Appendix 2 Numbers of records and individuals included in the machine learning model.

Multimedia Appendix 3 Association between chronic obstructive pulmonary disease and the top 30 variables of importance based on logistic regression.

Multimedia Appendix 4 Importance of each predictor in the XGBoost model (including questionnaire items).

## Abbreviations

5-CV: five times for cross-validation
AUC: area under the receiver operating characteristic curve
BD: bronchodilator
COPD: chronic obstructive pulmonary disease
EOS: eosinophil count
FEV1: forced expiratory volume in 1 second
FVC: forced vital capacity
Hb: hemoglobin
HT: hematocrit
MCHC: mean corpuscular hemoglobin concentration
MCV: mean corpuscular volume
WBC: white blood cell
XGBoost: Gradient Boosting Decision Tree machine learning method
