# Supplementary material for: Machine Learning Methods for the Diagnosis of Chronic Obstructive Pulmonary Disease in Healthy Subjects: Retrospective Observational Cohort Study
Source: JMIR Med Inform. 2021 Jul 6;9(7):e24796. doi: 10.2196/24796 (PMC8293159; doi:10.2196/24796)
Supplement: Multimedia Appendix 1 [file medinform_v9i7e24796_app1.docx]

#### Multimedia Appendix 1. Clinical assessments and questions used in the analysis, and list of variables

| **Type** | **Measurement and Unit** |
| --- | --- |
|  |  |
| Demographic | Smoking status, n |
|  | Sex, n |
|  | Age, years |
| Lung function test | FEV_1_, L |
|  | VC, L |
|  | FEV_1_/FVC, % |
|  | %VC, % |
|  | %FEV_1_, % |
| Vitals | BMI, kg/m^2^ |
|  | Body fat ratio, % |
|  | Diastolic blood pressure, mmHg |
|  | Systolic blood pressure, mmHg |
| Laboratory tests | ALP, U/L |
|  | BUN, mg/dL |
|  | AST, U/L |
|  | ALT, U/L |
|  | HbA1c, % |
|  | HDL-C, mg/dL |
|  | Hematocrit, % |
|  | Hb, g/dL |
|  | KET, n |
|  | LDL, mg/dL |
|  | MCH, pg |
|  | MCHC, g/L |
|  | MCV, fL |
|  | PLT, ×10^4^/µL |
|  | RBC, ×10^4^/µL |
|  | CRE, mg/dL |
|  | T-BIL, mg/dL |
|  | TC, mg/dL |
|  | Fasting blood sugar, mg/dL |
|  | TP, g/dL |
|  | U-CRE, mg/day |
|  | U-RBC, n |
|  | UA, mg/dL |
|  | Protein in urine, n |
|  | Urinary sugar, n |
|  | UPH, pH |
|  | U-WBC, n |
|  | WBC, ×10^2^ /µL |
|  | GGT, U/L |
|  | ALB, g/dL |
|  | CHE, U/L |
|  | Occult blood in urine, n |
|  | D-BIL, mg/dL |
|  | EOS, % |
|  | IRI, µU/mL |
|  | Urobilinogen in urine, n |
|  | SG, ratio |
|  | Pulse, times/min |
|  | CRP, mg/dL |
|  | eGFR, mL/min/1.73m^2^ |
|  | EOS_number, /mm^3^ |
| Questions about lifestyle habits | Average sleeping time in the past 1 month, hour |
|  | Number of alcohol consumption days per week, n |
|  | Amount of alcohol consumed per day, L |
|  | “I have breakfast everyday”, n |
|  | Walking time for commuting to work, hour |
|  | Physical activity at work, hour |
|  | Regular exercise, hour |
| Questions about symptom | “I have palpitation or disturbed pulse”, n |
|  | “I have chest compression and pain”, n |
|  | “I have cough and sputum”, n |
|  | “I have gastric distress and nausea”, n |
|  | “I have stiff neck or back pain”, n |
|  | “My fingers or arms are numb or painful”, n |
|  | “I have low back pain”, n |
|  | “My hip, thighs, or calf are numb or painful”, n |
| Questions about treatment history | “I take anti-hypertension drugs”, n |
|  | “I take insulin injection or anti-hyperglycemic drugs”, n |
|  | “I take anti-hyperlipidemic drugs”, n |
|  | “I am under treatment_other diseases”, n |
|  | “I am under treatment_epilepsy”, n |
|  | “I am under treatment_allergic diseases”, n |
|  | “I am under treatment_arrythmia”, n |
|  | “I am under treatment_insomnia”, n |
|  | “I am under treatment_duodenal ulcer”, n |
|  | “I am under treatment_colorectal polyp”, n |
|  | “I am under treatment_myocadiac infarction”, n |
|  | “I am under treatment_malignancy”, n |
|  | “I am under treatment_chronic hepatitis”, n |
|  | “I am under treatment_slipped disk”, n |
|  | “I am under treatment_bronchial asthma”, n |
|  | “I am under treatment_angina”, n |
|  | “I am under treatment_throid diseases”, n |
|  | “I am under treatment_psychiatric diseases”, n |
|  | “I am under treatment_diabetes”, n |
|  | “I am under treatment_tuberculosis”, n |
|  | “I am under treatment_stomach ulcer”, n |
|  | “I am under treatment_gallstone”, n |
|  | “I am under treatment_liver steatosis”, n |
|  | “I am under treatment_stroke”, n |
|  | “I am under treatment_kidney diseases”, n |
|  | “I am under treatment_anemia”, n |
|  | “I am under treatment_rheumatoid arthritis, collagen diseases”, n |
|  | “I am under treatment_hyperuricemia”, n |
|  | “I am under treatment_hyperlipidemia”, n |
|  | “I am under treatment_hypertension”, n |
|  | “I had surgery_lung”, n |
| Electrocardiogram (ECG) | ECG_trigeminal pulse, n |
|  | ECG_bigeminal pulse, n |
|  | ECG_PAC, n |
|  | ECG_PAC short run, n |
|  | ECG_PAC paired pulses, n |
|  | ECG_PAC frequent occurrence, n |
|  | ECG_SVT, n |
|  | ECG_inferior infarction, n |
|  | ECG_possible inferior infarction, n |
|  | ECG_suspected inferior infarction, n |
|  | ECG_Incomplete right bundle branch block, n |
|  | ECG_indeterminate axis, n |
|  | ECG_combined ventricular hypertrophy, n |
|  | ECG_bifascicular block, n |
|  | ECG_subacute anterior infarction, n |
|  | ECG_subacute anteroseptal infarction, n |
|  | ECG_artificial cardiac pacemaker rhythm, n |
|  | ECG_artificial cardiac pacemaker rhythm.A, n |
|  | ECG_artificial cardiac pacemaker rhythm.D, n |
|  | ECG_low voltage.limb lead, n |
|  | ECG_low voltage.chest lead, n |
|  | ECG_lateral infarction, n |
|  | ECG_possible lateral infarction, n |
|  | ECG_suspected lateral infarction, n |
|  | ECG_coronary vein sinus rhythm, n |
|  | ECG_anteroseptal infarction, n |
|  | ECG_possible anteroseptal infarction, n |
|  | ECG_susptected anteroseptal infarction, n |
|  | ECG_anterior infarction, n |
|  | ECG_possible anterior infarction, n |
|  | ECG_suspected anterior infarction, n |
|  | ECG_counterclockwise, n |
|  | ECG_right ventricular hypertrophy, n |
|  | ECG_right ventricular hypertrophy and right atrial enlargement, n |
|  | ECG_right ventricular hypertrophy and left atrial enlargement, n |
|  | ECG_right atrial enlargement, n |
|  | ECG_dextrocardia, n |
|  | ECG_right axis deviation, n |
|  | ECG_boundary Q wave, n |
|  | ECG_complete right bundle block, n |
|  | ECG_complete left bundle block, n |
|  | ECG_complete atrioventricular block, n |
|  | ECG_left ventricular hypertrophy, n |
|  | ECG_left ventricular hypertrophy and left atrial enlargement, n |
|  | ECG_suspected left ventrical hypertrophy, n |
|  | ECG_left atrial enlargement, n |
|  | ECG_suspected left ventricular rhythm, n |
|  | ECG_left anterior hemiblock, n |
|  | ECG_suspected left anterior hemiblock, n |
|  | ECG_left poterior hemiblock, n |
|  | ECG_left axis deviation, n |
|  | ECG_giant negative T, n |
|  | ECG_flat-low T, n |
|  | ECG_bradycardia, n |
|  | ECG_ventricular trigeminy, n |
|  | ECG_ventricular bigeminy, n |
|  | ECG_intraventricular conduction disorder, n |
|  | ECG_ventricular extrasystole, n |
|  | ECG_venticular extrasystole short run, n |
|  | ECG_ventricular extrasystole double pulse, n |
|  | ECG_ventricular extrasystole, frequent pulse, n |
|  | ECG_ventricular rhythm, n |
|  | ECG_atrial flutter, n |
|  | ECG_atrial fibrillation, n |
|  | ECG_acute inferior infarction, n |
|  | ECG_acute anterior infarction, n |
|  | ECG_suspected acute anterior infarction, n |
|  | ECG_atrioventricular block grade I, n |
|  | ECG_atrioventricular block grade II (Wenckebach), n |
|  | ECG_atrioventricular block grade II (Mobitz II), n |
|  | ECG_atrioventricular block grade II (2:1), n |
|  | ECG_ventricular extrasystole associated with atrioventricular block, n |
|  | ECG_atrioventricular junctional rhythm, n |
|  | ECG_atrioventricular dissociation, n |
|  | ECG_clockwise, n |
|  | ECG_normal range, n |
|  | ECG_sinus arrhythmia, n |
|  | ECG_sinus bradycardia, n |
|  | ECG_sinoatrial block, n |
|  | ECG_sinus tachycardia, n |
|  | ECG_abnormal Q wave, n |
|  | ECG_indeterminate arrhythmia, n |
|  | ECG_escaped beat, n |
|  | ECG_recording faults, n |
|  | ECG_mild right ventricular hypertrophy, n |
|  | ECG_mild left ventricular hypertrophy, n |
|  | ECG_mild left axis deviation, n |
|  | ECG_mild QT prolongation, n |
|  | ECG_mild right ventricular hypertrophy and right atrial enlargement, n |
|  | ECG_mild right ventricular hypertrophy and left atrial enlargement, n |
|  | ECG_mild left ventricular hypertrophy and left atrial enlargement, n |
|  | ECG_suspected mild abnormal ST-T, n |
|  | ECG_mild ST elevation, n |
|  | ECG_mild abnormal ST.T, n |
|  | ECG_negative T, n |
|  | ECG_electrode errot, n |
|  | ECG_tachycardia, n |
|  | ECG_high T wave, n |
|  | ECG_possible elevated posterior infarction, n |
|  | ECG_suspected elevated posterior infarction, n |
|  | ECG_severe right axis deviation, n |
|  | ECG_severe bradycardia, n |
|  | ECG_severe tachycardia, n |
|  | ECG_high voltage, n |
|  | ECG_coved mild ST elevation. right thorax, n |
|  | ECG_coved ST elevation. right thorax, n |
|  | ECG_J wave-associated ST elevation, n |
|  | ECG_P-R shortening, n |
|  | ECG_PR prolongation, n |
|  | ECG_QT prolongation, n |
|  | ECG_QT shortening, n |
|  | ECG_poor R progression, n |
|  | ECG_RSR pattern, n |
|  | ECG_S1 S2 S3 pattern, n |
|  | ECG_abnormal ST-T, n |
|  | ECG_ST elevation, n |
|  | ECG_ST elevation-associated right bundle branch block, n |
|  | ECG_saddleback ST elevation.right thorax, n |
|  | ECG_WPW syndrome, n |
|  | ECG_suspected WPW syndrome, n |
|  | ECG_suspected WPW syndrome A-type, n |
|  | ECG_suspected WPW syndrome B-type, n |
|  | ECG_suspected WPW syndrome C-type, n |
|  | ECG_WPW syndrome A-type, n |
|  | ECG_WPW syndrome B-type, n |
|  | ECG_WPW syndrome C-type, n |
| Computed tomography (CT) | CT_suspected pneumoconiosis = no, n (%)  CT_abnormal cardiac great blood vessel = yes, n (%)  CT_abnormal mediastinum.pulmonary hilum = yes, n (%)  CT_pulmonary mass = yes, n (%)  CT_abnormal pleura.parapet = yes, n (%)  CT_suspected diffusive lung disease = yes, n (%)  CT_bulla, bleb = yes, n (%)  CT_moderate emphysema= yes, n (%)  CT_middle mediastinum pulmonary mass= yes, n (%)  CT_abnormal mammary gland = yes, n (%)  CT_anterior mediastinum pulmonary mass = yes, n (%)  CT_aneurysm = yes, n (%)  CT_suspected primary lung cancer = yes, n (%)  CT_right coronary arterial calcification = yes, n (%)  CT_aortic calcification = yes, n (%)  CT_left anterior descending branch calcification = yes, n (%)  CT_left circumflex branch calcification = yes, n (%)  CT_posterior mediastinum pulmonary mass = yes, n (%)  CT_cardiac dilatation = yes, n (%)  CT_chronic inflammation = yes, n (%)  CT_photographing error = yes, n (%)  CT_tracheal.bronchial stenosis = yes, n (%)  CT_suspected active pulmonary tuberculosis = no, n (%)  CT_atelectasis = yes, n (%)  CT_thyroid mass = yes, n (%)  CT_not particular = yes, n (%)  CT_mediastinum.hilar lymph node calcification = yes, n (%)  CT_mediastinum.hilar adenopathy = yes, n (%)  CT_abnormal pulmonary area = yes, n (%)  CT_pleura plaque = yes, n (%)  CT_pleural callosity.pleurodesis.pleural calcification = yes, n (%)  CT_pleural.chest wall tumor = yes, n (%)  CT_post-thoracoscopy status = yes, n (%)  CT_benign tumor = yes, n (%)  CT_mild emphysema = yes, n (%)  CT_obsolete pulmonary tuberculosis = yes, n (%)  CT_osseous abnormalities = yes, n (%)  CT_severe emphysema = yes, n (%)  hasCT = true (%)  location_right upper lung field.upper lobe, n (%)  location_right lower lung field.lower lobe, n (%)  location_right middle lung field.middle lobe, n (%)  location_left upper lung field.segmentum superius, n (%)  location_left lower lung field.lower lobe, n (%)  location_left middle lung field.lingula, n (%)  location_whole lung field, n (%)  location_pulmonary hilum.mediastinum, n (%) |
| Bundled or calculated from questionnaires | Cough, n |
|  | Phlegm, n |
|  | Abnormal breathing, n |
|  | Allergic symptoms, n |
|  | Has treatment for lung or bronchus, n |
|  | Pack_year, pack/days × years |
|  | Smoking duration, years |
|  | Years from smoking cessation, years |

Abbreviations: ALB, albumin; ALP, alkaline phosphatase; ALT, serum alanine aminotransferase; AST, aspartate aminotransferase; BMI, body mass index; BUN, blood urea nitrogen; CHE, cholinesterase; CRE, creatinine; CRP, C-reactive protein; D-BIL, direct bilirubin; eGFR, estimated glomerular filtration rate; ECG, electrocardiogram; EOS, eosinophil count; FEV_1_, forced expiratory volume in 1 second; FVC, forced vital capacity; GGT, gamma-glutamyl transferase; Hb, hemoglobin; HbA1c, hemoglobin A1c; HDL-C, high-density lipoprotein cholesterol; IRI, insulin; KET, ketone bodies; LDL, low-density lipoprotein cholesterol; MCH, mean corpuscular hemoglobin; MCHC, mean corpuscular hemoglobin concentration; MCV, mean corpuscular volume; PAC, premature atrial contraction; PLT, platelet; RBC, red blood cell; SG, urine specific gravity; SVT, supraventricular tachycardia; T-BIL, total bilirubin; TC, total cholesterol; TP, total protein; UA, uric acid; U-RBC, red blood cell in urine; U-CRE, creatinine in urine; UPH, urine pH; U-WBC, white blood cell in urine; VC, vital capacity; WBC, white blood cell; WPW, Wolf–Parkinson–White.
