# Supplementary material for: Machine Learning Methods for the Diagnosis of Chronic Obstructive Pulmonary Disease in Healthy Subjects: Retrospective Observational Cohort Study
Source: JMIR Med Inform. 2021 Jul 6;9(7):e24796. doi: 10.2196/24796 (PMC8293159; doi:10.2196/24796)
Supplement: Multimedia Appendix 2 [file medinform_v9i7e24796_app2.docx]

**Multimedia Appendix 2.** **Numbers of records and individuals included in the machine learning model**

|  | **Number of records** | | | **Number of individuals** | | |
| --- | --- | --- | --- | --- | --- | --- |
|  | COPD | Non-COPD | Total | COPD | Non-COPD | Total |
|  |  |  |  |  |  |  |
| Train | 1,668 | 34,287 | 35,955 | 657 | 13,528 | 14,185 |
| Test | 767 | 14,710 | 15,477 | 297 | 5,783 | 6,080 |
| Total | 2,435 | 48,997 | 51,432 | 954 | 19,311 | 20,265 |

Abbreviation: COPD, chronic obstructive pulmonary disease
