# Supplementary material for: Machine Learning Methods for the Diagnosis of Chronic Obstructive Pulmonary Disease in Healthy Subjects: Retrospective Observational Cohort Study
Source: JMIR Med Inform. 2021 Jul 6;9(7):e24796. doi: 10.2196/24796 (PMC8293159; doi:10.2196/24796)
Supplement: Multimedia Appendix 3 [file medinform_v9i7e24796_app3.docx]

#### Multimedia Appendix 3. Association between chronic obstructive pulmonary disease and the top 30 variables of importance based on logistic regression

| **Variable** | **Importance of XGBoost model** | **Coefficient of logistic regression** |
| --- | --- | --- |
|  |  |  |
| FEV_1_/FVC | 0.231 | −0.374 |
| Smoking status | 0.030 | 0.295 |
| Allergic symptoms | 0.028 | −0.469 |
| Regular exercise | 0.027 | None^a^ |
| Pack_year | 0.023 | None^a^ |
| Cough | 0.022 | 0.083 |
| I have chest compression and pain | 0.019 | None^a^ |
| Average sleeping time in the past 1 month | 0.017 | 0.021 |
| HbA1c | 0.016 | −0.207 |
| MCV | 0.016 | 1.009 |
| ALB | 0.015 | −0.061 |
| %FEV_1_ | 0.015 | −1.674 |
| I have breakfast everyday | 0.015 | −0.228 |
| Smoking duration | 0.013 | None^a^ |
| Body fat ratio | 0.013 | None^a^ |
| Hb | 0.013 | 0.603 |
| Hematocrit | 0.013 | −0.265 |
| TP | 0.012 | −0.071 |
| %VC | 0.012 | 1.466 |
| High-density lipoprotein cholesterol | 0.012 | None^a^ |
| Age | 0.012 | None^a^ |
| MCHC | 0.011 | 0.110 |
| Allergic disease | 0.011 | −0.157 |
| EOS | 0.010 | None^a^ |
| MCH | 0.010 | −0.929 |
| Systolic blood pressure | 0.010 | None^a^ |
| Fasting blood sugar | 0.010 | None^a^ |
| Serum alanine aminotransferase | 0.010 | None^a^ |
| Diastolic blood pressure | 0.010 | None^a^ |
| BUN | 0.009 | None^a^ |

^a^Removed by recursive feature elimination in the training logistic regression model

Abbreviations: ALB, albumin; BUN, blood urea nitrogen; COPD, chronic obstructive pulmonary disease; EOS, eosinophil count; FEV_1,_ forced expiratory volume in 1 second; FVC, forced vital capacity; Hb, hemoglobin; HbA1c, hemoglobin A1c; MCH, mean corpuscular hemoglobin; MCHC, mean corpuscular hemoglobin concentration; MCV, mean corpuscular volume TP, total protein; VC, vital capacity.
