# Supplementary material for: Machine Learning Methods for the Diagnosis of Chronic Obstructive Pulmonary Disease in Healthy Subjects: Retrospective Observational Cohort Study
Source: JMIR Med Inform. 2021 Jul 6;9(7):e24796. doi: 10.2196/24796 (PMC8293159; doi:10.2196/24796)
Supplement: Multimedia Appendix 4 [file medinform_v9i7e24796_app4.docx]

#### Multimedia Appendix 4. Importance of each predictor in the XGBoost model (including questionnaire items)

| **Variable** | **Importance** |
| --- | --- |
| Forced Expiratory Volume in 1 second/Force Vital Capacity | 0.2313 |
| Smoking status | 0.0302 |
| Allergic symptoms | 0.0280 |
| Regular exercise | 0.0275 |
| Smoking Pack year | 0.0228 |
| Cough | 0.0216 |
| I have chest compression and pain | 0.0192 |
| Average sleeping time in the past 1 month | 0.0169 |
| Hemoglobin A1c | 0.0158 |
| Mean corpuscular volume | 0.0156 |
| Albumin | 0.0151 |
| % Forced Expiratory Volume in 1 second | 0.0148 |
| I have breakfast everyday | 0.0146 |
| Smoking duration | 0.0134 |
| Body fat ratio | 0.0132 |
| Hemoglobin | 0.0132 |
| Hematocrit | 0.0129 |
| Total protein | 0.0124 |
| % Vital Capacity | 0.0117 |
| High-density lipoprotein cholesterol | 0.0117 |
| Age | 0.0116 |
| Mean corpuscular hemoglobin concentration | 0.0106 |
| Allergic disease | 0.0106 |
| Eosinophil count | 0.0101 |
| Mean corpuscular hemoglobin | 0.0100 |
| Systolic blood pressure | 0.0100 |
| Fasting blood sugar | 0.0099 |
| Serum alanine aminotransferase | 0.0096 |
| Diastolic blood pressure | 0.0096 |
| Blood urea nitrogen | 0.0094 |
